# The Dynamics of Synthesis and Localization of Jumbo Phage RNA Polymerases inside Infected Cells

**DOI:** 10.3390/v15102096

**Published:** 2023-10-16

**Authors:** Daria Antonova, Viktoriia V. Belousova, Erik Zhivkoplias, Mariia Sobinina, Tatyana Artamonova, Innokentii E. Vishnyakov, Inna Kurdyumova, Anatoly Arseniev, Natalia Morozova, Konstantin Severinov, Mikhail Khodorkovskii, Maria V. Yakunina

**Affiliations:** 1 Research Center of Nanobiotechnologies, Peter the Great St. Petersburg Polytechnic University, St. Petersburg 195251, Russia; 2 Group of Molecular Cytology of Prokaryotes and Bacterial Invasion, Institute of Cytology of the Russian Academy of Science, St. Petersburg 194064, Russia; innvish@gmail.com; 3 Center for Precision Genome Editing and Genetic Technologies for Biomedicine, Institute of Gene Biology Russian Academy of Sciences, Moscow 119334, Russia; 4 Institute of Molecular Genetics National Kurchatov Center, Moscow 123182, Russia; 5 Waksman Institute for Microbiology, Rutgers, The State University of New Jersey, Piscataway, NJ 08854, USA

**Keywords:** jumbo phages, phiKZ, transcription regulation, phage nucleus, virion RNA-polymerase, giant phages, pseudo-nucleus

## Abstract

A nucleus-like structure composed of phage-encoded proteins and containing replicating viral DNA is formed in *Pseudomonas aeruginosa* cells infected by jumbo bacteriophage phiKZ. The PhiKZ genes are transcribed independently from host RNA polymerase (RNAP) by two RNAPs encoded by the phage. The virion RNAP (vRNAP) transcribes early viral genes and must be injected into the cell with phage DNA. The non-virion RNAP (nvRNAP) is composed of early gene products and transcribes late viral genes. In this work, the dynamics of phage RNAPs localization during phage phiKZ infection were studied. We provide direct evidence of PhiKZ vRNAP injection in infected cells and show that it is excluded from the phage nucleus. The nvRNAP is synthesized shortly after the onset of infection and localizes in the nucleus. We propose that spatial separation of two phage RNAPs allows coordinated expression of phage genes belonging to different temporal classes.

## 1. Introduction

During bacteriophage infection, the expression of viral genes is tightly regulated to ensure that viral proteins are produced at the time when they are needed. Much of this regulation occurs at the level of transcription of viral genes. For most phages, three temporal classes of genes can be defined: early, middle, and late, according to the time after the infection when their transcription begins. Products of early transcribed genes typically orchestrate the take-over of the host by the virus; middle genes typically encode enzymes involved in phage genome replication, and late genes encode virion and host lysis proteins.

While the tripartite strategy of phage gene expression is broadly applicable to most phages, it is accomplished in very different ways. Some phages rely on the host RNAP to transcribe their genes. In this case, phage-encoded proteins direct the host RNAP to appropriate promoters throughout the infection [1,2] and/or phage-encoded antitermination factors allow host RNAP to access specific groups of phage genes located downstream of terminators [3,4,5]. Prototypical examples of such strategies are bacteriophages T4 and λ, correspondingly. Some phages encode their own RNAPs. These enzymes are frequently the products of early genes transcribed by host RNAP from strong promoters and are responsible for middle and/or late viral transcription [2,6]. A phage-encoded inhibitor of the host enzyme commonly accomplishes a switch from host to viral RNAP transcription. A prototypical example of such strategy is bacteriophage T7 [6]. Another example is bacteriophage N4, which uses two viral single-subunit polymerases and a host polymerase for its genome transcription. One RNAP is injected inside the cell with phage DNA and transcribes early genes [7,8]. Another RNAP is encoded by early genes and transcribes middle genes [9,10]. Late N4 gene transcription is performed by host RNAP [11].

Transcription of jumbo bacteriophages similar to *P. aeruginosa* phage phiKZ is completely independent of host RNAP [12,13,14]. These phages encode two multisubunit RNAPs [12,13,14,15]. One RNAP is located in the virion (vRNAP) and must be injected into the host cell along with phage DNA. It transcribes early phage genes. The second RNAP, composed of early gene products, is not present in virions (nvRNAP) and recognizes promoters of late viral genes [15,16]. Some phiKZ genes belong to the middle transcription class. It is unknown which of the two viral enzymes is responsible for their transcription. Also, it is unknown how transcription between different classes of jumbo phage genes is switched.

Immediately after the phiKZ DNA injection, a round compartment (RC) appears in the cell [17]. RC contains phage DNA [17] and, presumably, co-injected virion proteins. Later in infection, a large nucleus-like structure is formed in the middle of the cell [17,18]. The phage nucleus (also referred to as pseudo-nucleus) consists of a proteinaceous shell formed by phage-encoded Chimallin (Gp54) protein [19,20,21] with replicating phage DNA located inside [17,18,20]. For phiKZ-related phage 201phi2-1, it was shown that some phage proteins required for transcription and replication, including two subunits of its nvRNAP, are also localized inside the nucleus [22].

In this work, we studied the dynamics and localization of phiKZ RNAPs in infected cells. Our results show that at the very beginning of infection, vRNAP co-localizes with injected phage DNA. The vRNAP accumulated throughout the infection is localized in infected cell cytoplasm and is excluded from the nucleus. In contrast, nvRNAP is concentrated inside the nucleus. We propose that such compartmentalization ensures coordinated expression of phage genes during the infection.

## 2. Materials and Methods

### 2.1. Bacteriophage, Bacterial Strain and Growth Conditions

The phiKZ phage lysate was prepared as described previously [17]. Phage was further purified using PEG8000 purification protocol [23].

The *Pseudomonas aeruginosa* strain PAO1 culture was grown in a LB medium at 37 °C. To prepare infected cell samples, a 10 mL aliquot of overnight culture was added to 1 L of fresh LB medium. Growth was continued until OD600 reached 0.6 and phiKZ lysate was added at a multiplicity of infection (MOI) of 10. Aliquots were withdrawn at selected time points and infection was terminated by the addition of 100 μg/mL chloramphenicol and rapid cooling in an ice water bath. Cells were harvested by centrifugation (5000× *g* at 4 °C for 5 min), flash-frozen and stored at −20 °C until use. The infection efficiency was checked by determining the number of colony-forming units just before the infection and 5 min post infection. Only cultures that contained less than 20% of surviving cells were used for further processing. For fluorescent microscopy experiments, PAO1 cells were transformed with the plasmid expressing fusion protein genes as described in [24] and selected on LB agar plates containing 100 μg/mL carbenicillin [24]. A single transformed colony was grown overnight at 37 °C in liquid LB medium supplemented with 100 μg/mL carbenicillin. The culture was diluted 1:100 in fresh LB medium containing carbenicillin, grown at 37 °C until OD600 reached 0.2 and induced with arabinose (final concentrations of 0.1 or 0.2%). Growth was continued at 30 °C until OD600 reached 0.6. At this point, culture aliquots were used for fluorescent microscopy experiments or for phage infection to obtain phage progeny containing fluorescent fusion proteins.

### 2.2. Cloning

All oligonucleotides used for cloning and/or PCR are listed in the Supplementary Table S1. Gene *180* was amplified from DNA purified from infected cell cultures collected 30 min post-infection and cloned into the pET28a plasmid polylinker between the NdeI and BamHI sites; the resulting plasmid was named pET28aHis-Gp180. The gene of the mCherry fluorescent protein was inserted into the pHERD20T plasmid according to the manufacturer’s protocol of NEBuilder^®^ HiFi DNA Assembly kit (New England Biolabs, Ipswich, MA, USA). The resulting plasmid was named pHERDmCh. To create pHERDgp55-mCh, PhiKZ gene *55* was amplified from the pET28aHis-gp55 plasmid encoding histidine-tagged Gp55 [25] and inserted into pHERDmCh using the Gibson cloning method. The gp180 gene with optimized codons for *P. aeruginosa* was synthesized by IDT (Coralville, IA, USA) and inserted into the pHERDmCh plasmid downstream of and in frame with the mCherry gene sequence using the NEBuilder^®^ HiFi DNA Assembly kit (New England Biolabs, Ipswich, MA, USA). The resulting plasmid was named pHERDmCh-gp180.

### 2.3. Protein Expression and Purification

Rosetta(DE3) *E. coli* cells were transformed with pET28aHis-gp55 or pET28aHis-gp180. Cultures were grown to OD600 = 0.5–0.7 and expression of target genes was induced by the addition of 1 mM IPTG. Induced cultures continued to grow for 5 h out at 15 °C (for Gp55) or 22 °C (for Gp180). Lowering the temperature allowed us to obtain soluble recombinant proteins that formed inclusion bodies when overexpressed at 37 °C. Next, 2 g of wet biomass was disrupted by sonication in 20 mL of buffer A (40 mM Tris-HCl, pH 8.0, 10% glycerol, 500 mM NaCl, 1 mM DTT, 5 mM imidazole) followed by centrifugation at 11,000× *g* for 30 min. Clarified lysate was loaded onto a HisTrap HP 5 mL (GE Life Sciences, Marlborough, MA, USA) column equilibrated with buffer A and washed by the same buffer. The recombinant proteins were eluted with buffer B (buffer A with containing 250 mM imidazole). The eluted fractions were concentrated on an Amicon Ultra-4 Centrifugal Filter Unit with Ultracel-10 membrane (EMD Millipore, Burlington, MA, USA) and further purified by size exclusion chromatography using a Superdex 200 Increase 10/300 (GE Life Sciences, Marlborough, MA, USA) in TGED buffer (20 mM Tris–HCl pH 8.0, 5% glycerol, 0.5 mM EDTA, 1 mM DTT) with 200 mM NaCl. Protein concentration was determined using the Bradford assay.

### 2.4. Western Blotting

Purified His-Gp55 and His-Gp180 samples were provided to Rusbiolink, Russia for immunization of rats. Immune antisera were used as primary antibodies at 1:1000 dilution. Peroxidase Goat Anti-Rat IgG (Jackson ImmunoResearch, West Grove, PA, USA) was used as secondary antibody. Peroxidase activity was detected with SuperSignal™ West Pico Chemiluminescent Substrate (Thermo Scientific, Waltham, MA, USA). For mCherry and mCherry-fused protein detection, rabbit anti-tRFP antibodies (Evrogen, Moscow, Russia) were used as primary and Goat anti-rabbit IgG-peroxidase conjugate (Sigma Aldrich, St. Louis, MO, USA) as secondary antibodies. Western blotting was performed using Amersham Protran 0.45 NC nitrocellulose membranes (GE LifeScience, Marlborough, MA, USA).

To test antisera cross-reactivity, Western blotting was performed with PEG-purified phiKZ virions (10^9^ plaque-forming units) and 0.5 μg purified native nvRNAP (Figure S1a). To analyze the vRNAP and nvRNAP subunits synthesis dynamics, three independent cell infection experiments were performed. For each experiment, 100 μL of cell culture before the infection, and 5, 10, 15, 20, 30 and 40 min post-infection, was used. After probing with the anti-Gp180 sera, nitrocellulose membranes were stripped according to the manufacturer’s protocol and re-probed with the Gp55 antisera. To estimate Gp180 amounts in virions and in 30 min infected cells, dilutions of purified His-Gp180 of known concentration were used as calibrants. Blots were scanned using the ChemidocTM XRS+ system (Bio-Rad, Hercules, CA, USA), and individual band densities were measured and compared using Quantity One 1-D analysis software (Bio-Rad, Hercules, CA, USA). To plot Gp180 and Gp55 protein accumulation during infection, the values for each protein band density were normalized to maximal values.

### 2.5. Co-Immunoprecipitation

All procedures were performed at 4 °C. Cell pellets from 200 mL of infected cultures were resuspended in 1 mL of buffer C (40 mM Tris-HCl pH 8.0, 150 mM NaCl, 0.5 mM EDTA, 10% glycerol, 1 mM PMSF) and disrupted by sonication. Crude extracts were clarified by centrifugation at 10,000× *g* at 4 °C for 30 min. A total of 10 μL of Anti-mCherry ABs solution (Abcam, Waltham, MA, USA) was added and Eppendorf tubes containing lysates were left for 1 h on a platform of rotary shaker with gentle (100 rpm) agitation at 4 °C. The contents were next transferred to Micro Bio-spin columns (Bio-Rad, Hercules, CA, USA) containing 250 μL of A-sepharose (GE LifeScience, Marlborough, MA, USA) equilibrated with buffer C. The column was incubated for 1 h on a platform of rotary shaker with gentle agitation at 4 °C. The column was washed with 10 mL of buffer C and next with 3 mL of PBS buffer. Bound proteins were eluted with 250 μL of elution buffer (0.2% SDS, 0.1% Tween-20) and eluted proteins were precipitated with trichloroacetic acid, resuspended in Laemmli’s buffer and resolved by 10% SDS-PAGE.

### 2.6. Mass Spectrometry Analysis

Protein bands of interest were manually excised from the Coomassie-stained SDS–polyacrylamide gels. Individual slices were prepared for mass spectrometry by in situ trypsin digestion at 37 °C for 4 h as previously described [15].

### 2.7. Fluorescence Microscopy

Cells were withdrawn from infected cultures and immediately placed on microscopic slides, prepared as described in [26]. In the case of fusion protein expression, agarose pads contained 0.2% (mCh-Gp180) or 0.1% (Gp55-mCh) arabinose. When cells were absorbed, the agarose pad was sealed by a cover glass and VALAP (lanolin, paraffin, and petrolatum 1:1:1). Fluorescence microscopy was performed using a Nikon Eclipse Ti-E inverted microscope equipped with a custom incubation system. During microscopy, the incubation temperatures were 30 °C for Gp55-mCh expressing cells and 37 °C for other cells. Optimal concentrations of inducers and growth temperatures were determined empirically to avoid aggregation of recombinant proteins at standard induction conditions (0.2% arabinose and 37 °C). Fluorescence signals in DAPI and mCherry fluorescence channels were detected using Semrock filter sets DAPI-50LP-A and TxRed-4040C, respectively. Imaging was performed using Micro-Manager [27] with a custom script. Images were taken every 10 min for 180 min. Image analyses were performed using the ImageJ software.

The fluorescence intensity corresponding to a single mCherry molecule was calculated as described in [26]. First, the fluorescence of pure mCherry protein solution with known concentration was measured using a spectrofluorometer and intensity corresponding to a single mCherry molecule was calculated. Next, fluorescence of an aliquot of a culture containing a known number (CFUs) of *E. coli* cells producing mCherry was measured using a spectrofluorometer and the average number of fluorescent protein molecules per cell was determined. Individual cells from the same culture were next observed under the fluorescent microscope. Average fluorescence intensity of single cells was divided by the estimated average number of mCherry molecules per cell. In this way, the fluorescence intensity of a single mCherry molecule was obtained under the imaging conditions used. Since the imaging parameters such as exposure, the presence of filters, etc., differed between experiments, calibration coefficients were obtained for each imaging condition. These coefficients were calculated by determining the average fluorescence intensity of at least 400 mCherry-producing *E. coli* cells at a given imaging condition.

## 3. Results

### 3.1. Dynamics of phiKZ RNAPs Accumulation during Infection

**Figure 1:**
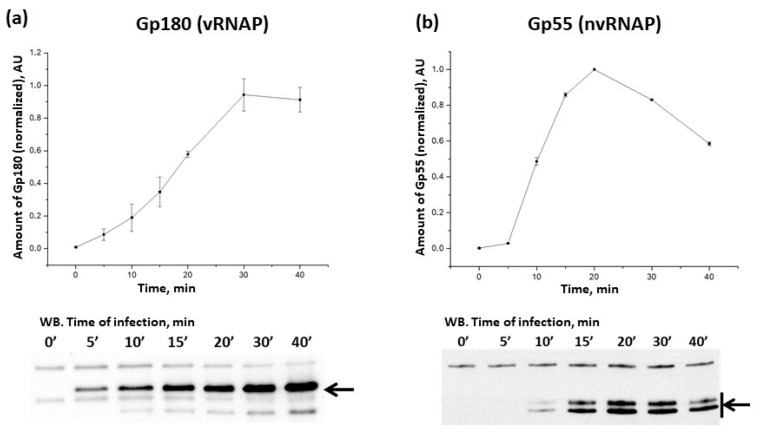
Accumulation of PhiKZ Gp180 and Gp55 in the course of the infection. Changes in RNAP subunit amounts (determined by the intensity of bands on Western blots such as the ones shown below) are presented. For each time point, means from three independent measurements are plotted; error bars represent standard deviations. Black arrows indicate the positions of Gp180 (**a**) and Gp55 (**b**) bands; Gp55 migrates as two bands.

To analyze the dynamics of phiKZ RNAP accumulation during infection, we used sera containing polyclonal antibodies (ABs) specific to the Gp180 subunit of vRNAP and Gp55 of nvRNAP (Figure S1a). Both subunits contain the universal Mg^2+^-binding domain DFDGD, which is essential for RNAP activity [28,29] and thus serves as a good proxy for measuring the amounts of active viral RNAPs. The number of vRNAP molecules in the virion was estimated to be 10 (±3) molecules based on the results of semiquantitative analysis using recombinant Gp180 with known concentration for calibration (Figure S1b). Gp180 was detected inside the cells 5 min post-infection and its amount steadily increased at later time points up to 30 min post-infection and remained constant afterwards (Figure 1a; note that infected cells start to lyse after 50 min post-infection). The subunits of vRNAP are the products of middle viral genes [12] and the profile of Gp180 accumulation follows the temporal profile of middle gene expression. The number of Gp180 molecules 30 min post-infection was estimated as ~3000 per cell by semi-quantitative Western blotting (Figure S1c). Given the reported phiKZ burst size of 100 progeny phage particles per cell [30], this result provides an upper estimate of ~30 vRNAP molecules per virion (note that not all vRNAP molecules may be packaged).

The nvRNAP Gp55 appeared 10 min post infection; its amount reached a maximum 20 min post infection and decreased at later times (Figure 1b). The pattern of Gp55 accumulation is consistent with the temporal profile of early gene expression, which encode, among other things, nvRNAP subunits [12].

### 3.2. Determination of Localization of Phage RNAPs during Infection Using Fluorescence Microscopy

**Figure 2:**
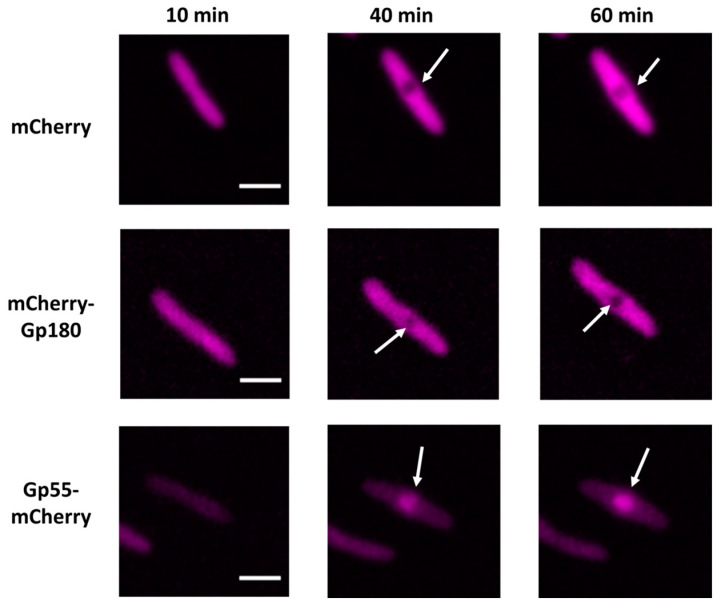
Localization of mCherry fluorescent protein, and Gp180 and Gp55 mCherry fusions during the PhiKZ infection. White arrows point to the phage nucleus (see also Figure S3). Times post-infection are indicated. All scale bars are 2 μm.

To monitor the intracellular localization of phage RNAPs within infected cells, we constructed expression plasmids carrying genes encoding phage RNAP subunits fused with the mCherry fluorescence protein (pHERD20TmCherry-gp180 and pHERDStr1gp55-mCherry, see Materials and Methods). Using Western blots, we showed that (1) both fusion proteins were produced by *P. aeruginosa* cells transformed with either of these plasmids, and (2) there was no degradation of fused proteins in either uninfected or infected cells (Figure S2). Live fluorescent microscopy revealed that both fusion proteins and the control mCherry protein were diffusely distributed in uninfected cells. In infected cells, mCherry and mCherry-Gp180 were localized in the cytoplasm and were absent from the phage nucleus. In contrast, mCherry-Gp55 accumulated inside the nucleus (Figure 2 and Figure S3).

**Figure 3:**
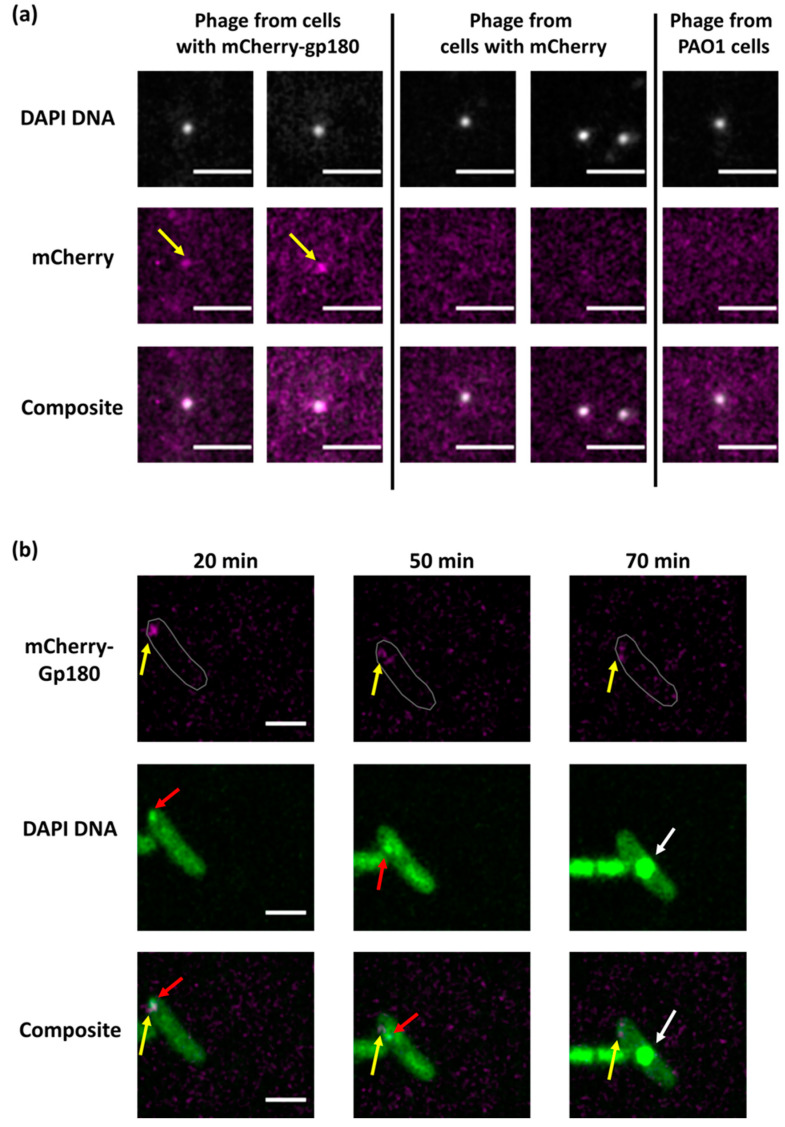
Localization of PhiKZ vRNAP in infected cells. (**a**). Co-localization of DAPI-stained DNA and the mCherry signals in PhiKZ phage particles in the lysate of mCherry-gp180-containing cells revealed by fluorescent microscopy. The scale bars are 1 μm. Phage particles from the lysate of mCherry-containing and native PAO1 strain cells are shown as controls. (**b**). Images of PAO1 cells infected with mCherry-Gp180-containing phiKZ phage. Yellow arrows point to mCherry-Gp180, red arrows point to the area of concentrated DNA, and white arrows to phage nucleus. Times indicate minutes post infection. In the top row, cell contour is delineated in gray. The scale bars are 2 μm.

Since mCherry-Gp180 localized like the mCherry control throughout the infection, it was necessary to show that its distribution reflects the localization of vRNAPs. We conducted immunoprecipitation with mCherry antibodies using extracts of infected and uninfected cells. In the infected cell sample, we detected the vRNAP subunits Gp178, Gp80 and Gp149 along with mCherry-Gp180 (Figure S4a), indicating that the fused subunit is indeed assembled into the vRNAP complex. We also collected phage progeny formed after the infection of mCherry-Gp180-producing cells and analyzed the protein content of progeny virions using Western blotting (Figure S4b) and fluorescent microscopy (Figure 3a). As a control, we used phages released from cells expressing the mCherry protein. The results showed that mCherry-Gp180 was incorporated into virions. Earlier, the coefficients for conversion of fluorescence intensities of individual cells to the number of mCherry fluorescent protein molecules were determined [26]. We used this information and fluorescence data for 15 virions to estimate the number of mCherry-Gp180 molecules per virion. Our measurements provided an estimate of 7 ± 4 molecules per particle (Figure S5), which is consistent with the number obtained using semi-quantitative Western blotting (above). This estimate should be considered as a lower boundary since non-fluorescent vRNAPs with Gp180 encoded by the phage may also be present in the virions.

Next, we used phages containing mCherry-Gp180 to infect *P. aeruginosa* cells and followed the infection using live fluorescence microscopy. As is shown in Figure 3b, the mCherry signal appeared in infected cells early in infection and co-localized with the bright DAPI-stained area corresponding to the round compartment, which contains injected phage DNA [17]. No mCherry signal was detected inside of the phage nucleus which formed in the middle of the cell later in infection. We conclude that injected vRNAP co-localizes with injected phage DNA at the earliest stages of infection and that both injected and newly synthesized vRNAP are excluded from the phage nucleus.

## 4. Discussion

In this work, we show using several independent methods that the number of vRNAP molecules per phiKZ virion is between 7 and 30, which is comparable to the number of early phage promoters [12,31]. We also show that phiKZ vRNAP is injected into infected cells and co-localizes with the phage genome in the round compartment (RC) at the earliest stages of infection [17], which must help compartmentalize early transcription and make it more efficient. The products of early genes are needed for phage nucleus formation and also encode the nvRNAP subunits. Once the injected PhiKZ genome finds itself in the nucleus, it starts to be replicated [17]. We show that nvRNAP is localized in the phage nucleus, where it must perform transcription of non-early viral genes. In contrast, both the initially injected and the much more abundant vRNAP synthesized late in infection are excluded from the phage nucleus. Compartmentalization of nvRNAP within and exclusion of vRNAP from the phage nucleus provides an elegant and simple mechanism of switch from early phage transcription.

Transcriptional profiling of phiKZ-infected cells reveals two classes of non-early transcripts: middle and late [12]. The nvRNAP transcribes late PhiKZ genes and recognizes promoters with a simple TATG consensus sequence, with G defining the transcription start point, both in vivo and in vitro [15]. Middle PhiKZ transcripts contain a conserved element upstream of their 5′ ends that is distinct from either early (vRNAP), late (nvRNAP), or host RNAP promoter consensus sequences [12]. Judging by their accumulation profiles, the middle transcripts must be produced within the phage nucleus. It therefore follows that nvRNAP is responsible for middle transcription. Since purified phiKZ nvRNAP holoenzyme transcribes from late promoters only [15], it follows that the recognition of middle promoters should require additional DNA- or nvRNAP-binding factors that remain to be identified. An alternative possibility is that middle transcripts are generated by processing of the early transcripts by a host or viral endoribonuclease that remains to be defined.

Additional vRNAP molecules are assembled throughout the infection upon translation in the cytoplasm. The newly synthesized vRNAP remains in the cytoplasm, solving yet another problem of prevention of early transcription from progeny phage genomes that accumulate in the nucleus. Eventually, these vRNAPs must be packaged into progeny phage particle heads that are assembled near the cell wall and then transferred to the cytoplasmic face of the nucleus to be packed with phage DNA [32]. Gp219, a protein of unknown function that we found to be associated with vRNAP in infected cells (Figure S4), is absent from the phiKZ virions [33,34] but may play a role in vRNAP packaging.

Overall, our results provide evidence that the jumbo phage nucleus allows, in addition to avoiding host defense mechanisms [35], to spatially separate transcription by two phage-encoded RNA polymerases, thus ensuring temporal coordination of phage transcription. Some jumbo phages do not encode homologs of the Chimallin protein that is the major component of the nucleus shell [18,20,36] and develop without the formation of a nucleus compartment in infected host cells. Invariably, the DNA of such jumbo phages contains uracyl instead of thymine, which, presumably, also allows it to avoid host defenses [13,37]. Uracyl-containing jumbo phages also encode two multisubunit RNAPs that are similar to nucleus-forming phage transcription enzymes [13,29]. Just like nucleus-forming phages, uracyl-containing phages rely on their vRNAP for early gene transcription and nvRNAP for later transcription (there are no middle promoters). Coordinated transcription of these uracyl-containing phage genomes is achieved in the absence of separate compartments and must therefore rely on specific mechanisms which remain to be determined.

## Data Availability

Data is contained within the article or Supplementary Material.

## Supplementary Materials

The following supporting information can be downloaded at: https://www.mdpi.com/article/10.3390/v15102096/s1, Figure S1: (a) Antibodies (ABs) raised against vRNAP His-Gp180 and nvRNAP His-Gp55 are specific and not cross-reactive with non-cognate PhiKZ RNAPs; (b) A representative Western blot used to semi-quantitatively estimate vRNAP amounts in virions; (c) A representative Western blot used to estimate vRNAP amounts inside infected cells; Figure S2: The mCherry fusions with nvRNAP Gp55 (a) and vRNAP Gp180 (b) are stable throughout the PhiKZ infection; Figure S3: Redistribution of Gp55-mCherry inside infected cell during infection; Figure S4: The mCh-Gp180 fusion is incorporated in vRNAP and progeny phage virions; Figure S5: Representative examples of individual phage particles with the mCherry-Gp180 fusion protein inside. Table S1: The list of oligonucleotides used for cloning.
