# Supplementary material for: The Dynamics of Synthesis and Localization of Jumbo Phage RNA Polymerases inside Infected Cells
Source: Viruses. 2023 Oct 16;15(10):2096. doi: 10.3390/v15102096 (PMC10612078; doi:10.3390/v15102096)
Supplement: Supplementary file 1 [file viruses-15-02096-s001.zip › viruses-2620111-supplementary.pdf]

Supplementary materials

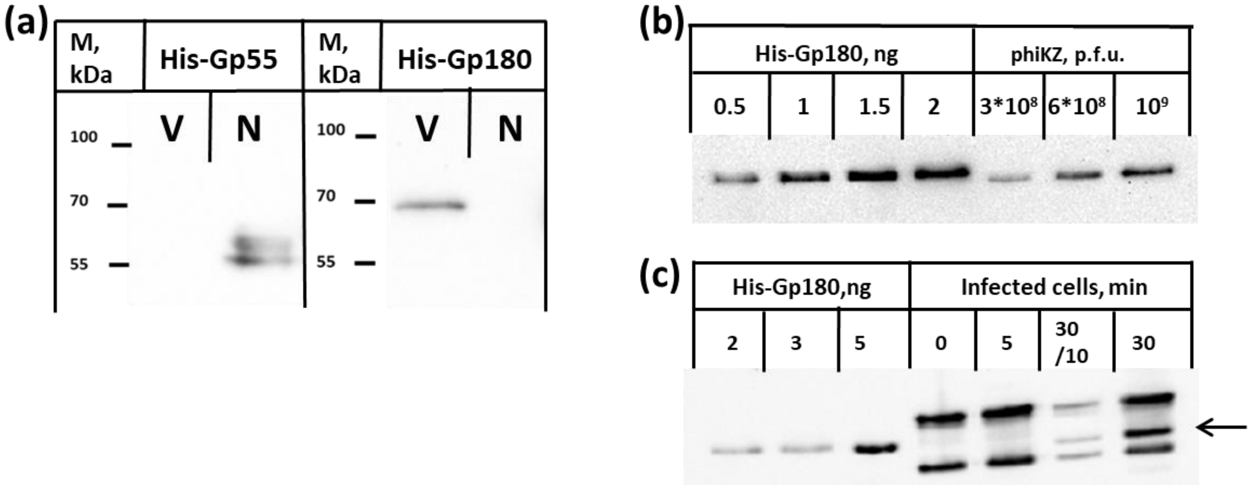

Figure S1: (a) Antibodies (ABs) raised against vRNAP His-Gp180 and nvRNAP His-Gp55 are specific and not cross-reactive with non-cognate PhiKZ RNAPs. Purified phiKZ virions were used as a sample containing vRNAP only (labeled "V"). Purified nvRNAP isolated from PhiKZ-infected cells was used as a sample containing nvRNAP only (labeled "N"). Proteins in both samples were separated by SDS PAGE and probed with antisera raised against recombinant vRNAP His-Gp180 or nvRNAP His-Gp55. (b) A representative Western blot used to semi-quantitatively estimate vRNAP amounts in virions. The right three lanes show signals obtained with samples containing indicated numbers of infectious PhiKZ virions (plaque forming units). Four left lanes show signals from indicated amounts of pure recombinant hexahistidine-tagged Gp180. (c) A representative Western blot used to estimate vRNAP amounts inside infected cells. Three lanes on the left show signals from indicated amounts of pure recombinant hexahistidine-tagged Gp180. Four lanes on the right show signals obtained with extracts of cells infected at a multiplicity of 2 and collected at indicated times post-infection. Cross-reacting bands above and below the Gp180 bands are present in both uninfected and infected cells and are coming from unidentified cell proteins. The two rightmost lanes show Gp180 signals from the same volume of 30-minute post-infection extract as used in other lanes (rightmost lane) and from the same volume of 10-fold diluted extract (second from right lane).

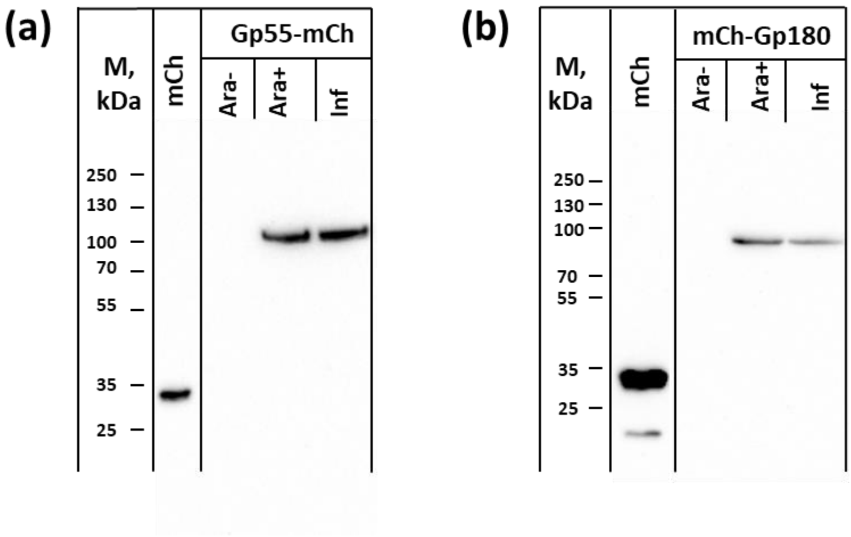

Figure S2. The mCherry fusions with nvRNAP Gp55 (a) and vRNAP Gp180 (b) are stable throughout the PhiKZ infection. Results of Western blotting with antibodies specific mCherry protein are shown. "mCh" – recombinant mCherry protein used as a control. In lanes labeled "Ara-" proteins from cells containing expression plasmids with indicated fused protein gene grown in the absence of arabinose inducer were separated. In "Ara+" lanes proteins from cells induced immediately prior to infection with PhiKZ were separated. In "Inf" lanes proteins from induced cells infected with PhiKZ at high MOI and collected 30 minutes post-infection were separated.

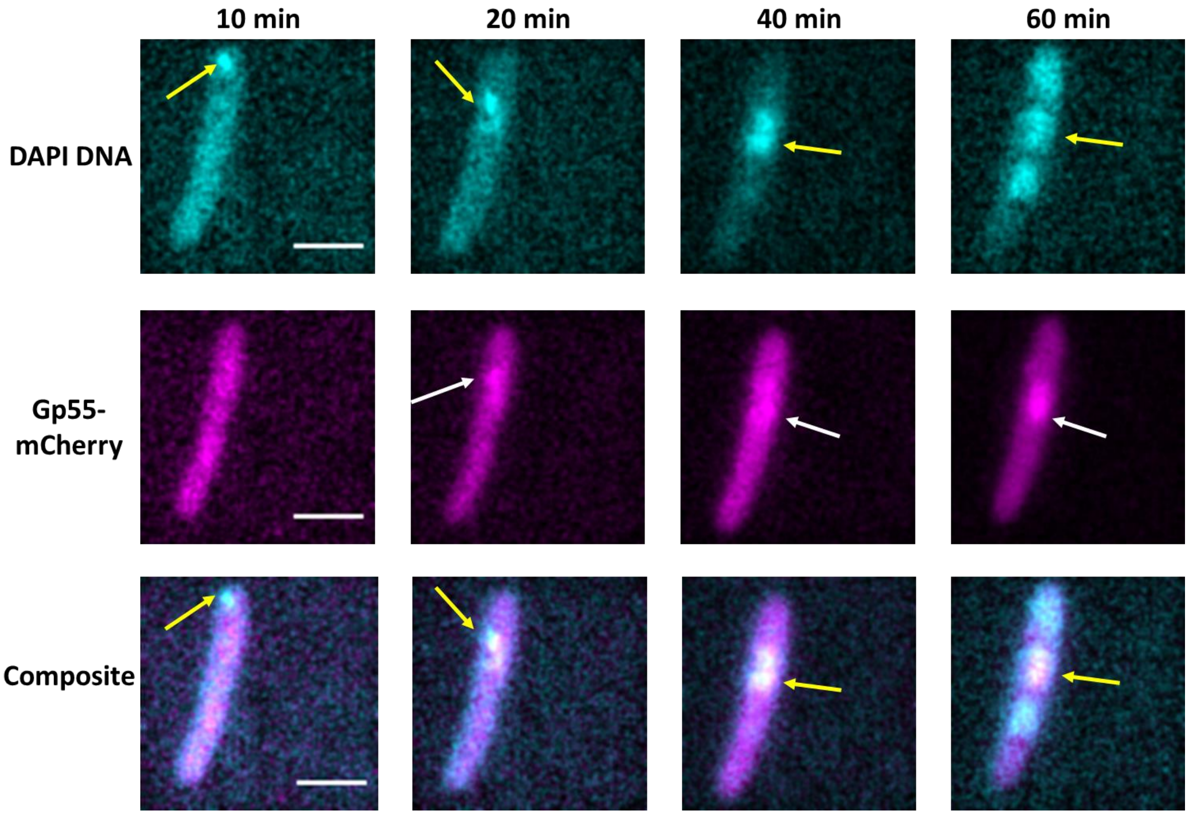

Figure S3: Redistribution of Gp55-mCherry inside infected cells during the infection. Yellow arrows point to areas of increased DNA concentration (first RC then phage phage nucleus). White arrows indicate an area of increased mCherry fluorescence. Times post-infection when images were taken is indicated at the top. The scale bars are 2 μm.

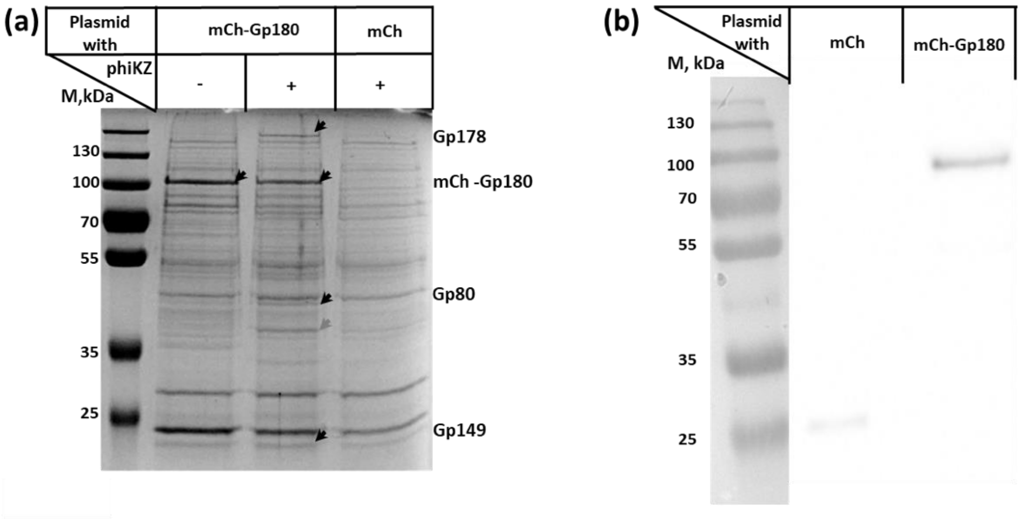

Figure S4: The mCh-Gp180 fusion is incorporated in vRNAP and progeny phage virions. (a) The result of co-immunoprecipitation with anti-mCherry antibodies from non-infected *P. aeruginosa* cells expressing mCherry-Gp180 fusion or phiKZ-infected cells expressing the mCherry-Gp180 fusion or mCherry alone ("mCh") collected 30 min post-infection. Protein bands enriched in cells expressing mCh-Gp180 with or without infection were analyzed by mass-spectrometry. Black arrows point to protein bands identified on the right. The grey arrow indicates Gp219, an early PhiKZ protein of unknown function. (b) Western-blot analysis of phiKZ progeny formed after infection of cells expressing mCherry or the mCh-Gp180 fusion. Material corresponding to 10^9 plaque forming units was loaded on each lane. The weak signal from the mCherry protein in the control (mCh) sample is attributed to either spurious incorporation of mCherry into virions or its binding to virions' surfaces.

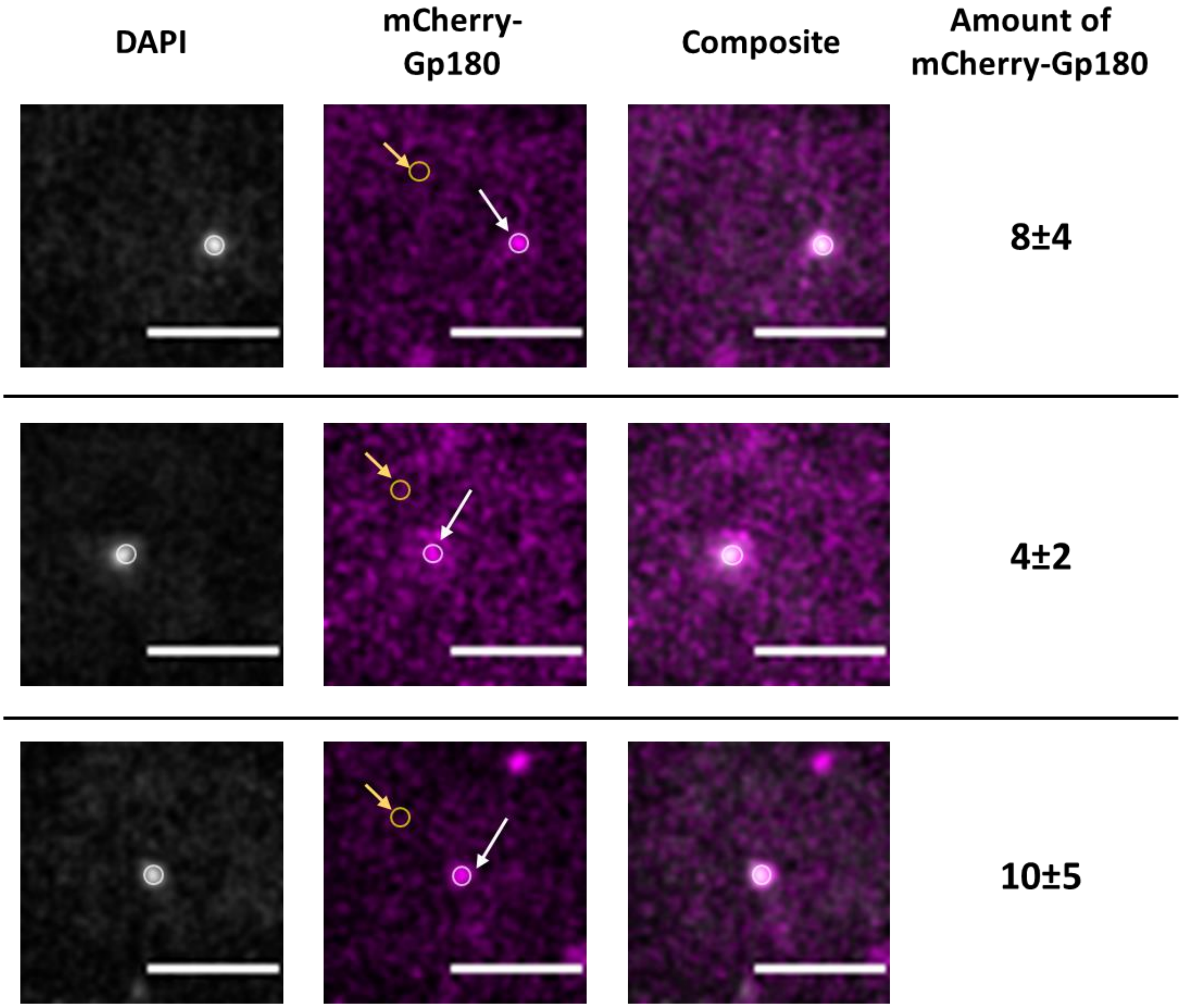

Figure S5: Representative examples of individual phage particles with the mCherry-Gp180 fusion protein inside. Phage particles formed after infection of mCherry-Gp180 overproducing cells were first imaged in the DAPI fluorescence channel, and 60x60 pixels squares containing individual phage particles were selected and duplicated. An area matching with a virion in the DAPI fluorescence channel (white circles, left) was selected by the oval selection tool and fluorescence intensities in the red channel (white circles and white arrows, middle) were measured. To measure background intensity, fluorescence was measured in the same-sized area without phage particles. Positions of background measurements are marked with yellow circles and yellow arrows in red fluorescence channel images (middle). Background intensity was subtracted from mCherry-Gp180 fluorescence intensity. Estimated numbers of mCherry-Gp180 molecules for each virion are presented for each of the three representative virions shown. The scale bars are 2 μm.

Table S1. The list of oligonucleotides used for cloning.

**Oligonucleotide name**	**Sequence**
pHERD20TG-F	GGGTATGTATATCTCCTTCTTAAAG
pHERD20TG-R	CACGTGCCATGGGATCTGATAAG
pHERD20TGp180Opt-GR	ATGAAGAAAGCGCTGGTGCC
mCherryG-R	CTTATCAGATCCCATGGCACGTGCTTGTACAGCTCGTCCATGC
mCherryG-F	CTTTAAGAAGGAGATATACATACCCATGGTGAGCAAGGGCGAGG
KZ180-F	AGGCATATGAAGAAGGCATTAGTTCC
KZ180-R	CTGGATCCTTAATCCCTTTGTTTGG
pHerdt-mCh-GR1	TCGGTACCCGGGGATCCTC
KZGp55F-G	AAGGAGATATACATACCCATGGGTCTTTATGCCAAAG
KZGp55-RminuSt-G	GAGGATCCCCGGGTACCGATTTTGCTTTTAGCCATTCTTC
